# Development and validation of a multi-modal contactless sensing system for surgical risk analysis in a real-world environment

**DOI:** 10.1371/journal.pdig.0001053

**Published:** 2025-11-13

**Authors:** Joseph R. Scarpa, Nidhi Kanchumarthi, Iqram Hussain, Aakash Keswani, Julianna Zeepvat, Andrew Milewski, Julia Scarpa, Richard Boyer, Rodrigo Sarlo

**Affiliations:** 1 Department of Anesthesiology, Weill Cornell Medicine, New York, New York, United States of America; 2 Department of Civil and Environmental Engineering, Virginia Polytechnic Institute and State University, Blacksburg, Virginia, United States of America; Rice University, UNITED STATES OF AMERICA

## Abstract

Gait measurements are a central component of functional assessments and risk stratification before surgery. Various sensors can measure gait metrics, but none are routinely integrated into surgical workflows because they are too challenging to implement at scale in clinical situations. In this manuscript, we report the development and validation of a rapidly-deployable, low footprint, entirely contactless sensing system, called GroundCode, that is explicitly integrated within a surgical workflow. GroundCode combines the Microsoft Kinect with seven floor-mounted single-axis accelerometers, overcoming the weaknesses of each individual sensor technology and providing both robust spatiotemporal resolution (Kinect) and high-fidelity footstep detection and quantification (floor accelerometers). We show that GroundCode-derived gait speed and cadence are highly precise measurements (>90%), and we validate them against two standard clinical gait measurements relevant to pre-surgical evaluations – stopwatch time and six-minute walk test distance. We show that GroundCode-derived gait metrics identify various surgical risk factors, like age, sex, and frailty. In addition, we show that preoperative gait is associated with postoperative quality of recovery. Importantly, we designed this system to be deployed by non-technical personnel and performed this study in a non-laboratory setting, providing proof-of-principle that GroundCode can be used in various real-world environments. We conclude that GroundCode provides highly robust gait measurements in real-world settings with possible applications spanning clinical diagnosis, risk stratification, and digital biomarker development.

## Author summary

Wearables and other sensors offer great promise for monitoring patients both inside and outside of the hospital. But these technologies are often difficult to integrate into clinical workflows because they need technical support, patient compliance, and standardized implementation protocols. To address these limitations, we created a contactless sensor system, called GroundCode, that transforms a room into a continuous health sensor. This facilitates its integration into a surgical workflow for preoperative risk assessment. Our sensing system can be set up in under 10 minutes in any environment and makes highly accurate measurements of how someone walks and moves. This technology has applications for disease diagnosis, clinical risk prediction, and at-home health monitoring, and has the potential to facilitate aging-in-place and other healthcare delivery models that prioritize patient well-being and recovery outside of the hospital.

## Introduction

Gait-based functional assessments are used to risk stratify individuals before surgery and to predict morbidity and mortality after surgery [1–4]. Various gait parameters – like speed and cadence – can be measured by wearables, cameras and sensors. Wearables allow for remote longitudinal monitoring in numerous clinical and non-clinical contexts and capture multiple features in addition to gait, providing information about a patient’s physiology outside of the hospital. Vicon, a marker-based motion capture system, measures gait biomechanics with great precision using specialized camera tracking of markers placed on various body parts. Specialized walkways, like GAITRite, also capture accurate temporal and spatial gait metrics, utilizing a mobile mat with a high density grid of pressure sensors that can be used in various contexts. Despite the development of these technologies, clinical gait testing remains the standard-of-care in healthcare and includes quantitative gait assessments (e.g., stopwatch-based gait speed and six-minute walk test) and qualitative gait descriptions (e.g., “hypokinetic”, “ataxic”, “Parkinsonian”). Only clinical assessments of gait are ever incorporated into pre-surgical evaluations.

Even though gait-based functional assessments are important for pre-surgical risk evaluation, wearables, specialized sensors, and even clinical assessments have several drawbacks that prevent their routine and widespread integration into surgical workflows. Clinical evaluation is time-intensive, inconvenient for the patient, and requires expert assessment. Wearable and other specialized devices are limited by variable accuracy [5] and require high quality end-user experience, technical support, and patient compliance for successful healthcare implementation [6]. In addition, specialized devices need operational infrastructure to facilitate their integration into clinical practice. These drawbacks prevent their widespread implementation in surgical workflows, which need to be optimized to evaluate high volumes of patients efficiently in order to maximize healthcare access and deliver high-quality care.

In light of these significant challenges, contactless sensors have emerged as a potential solution because they can be integrated directly into clinical environments and workflows [7]. Recent advances in machine learning and sensor technology have made feasible ambient intelligence systems, comprising sensors that continuously monitor patients in a physical space without the need for a monitor physically attached to the patient [7]. With contactless sensing, a physical space can be repurposed to sense and measure aspects of human health and physiology, removing barriers to access like technology literacy and cognitive health. Ambient intelligence systems provide a highly flexible framework that can be applied in various inpatient, outpatient, and home environments.

Contactless sensors have been used in various contexts to identify digital biomarkers for health and disease [8–13]. The Microsoft Kinect is one such ambient sensor that quantifies gait characteristics to predict frailty [14], Alzheimer’s disease [15], Parkinson’s disease severity [16], and mood [17–19]. The Kinect successfully classifies these diseases through highly accurate measurements at proximal joints (like pelvis, hip, and knee joints). However, the Kinect has poor accuracy when measuring foot and ankle joints [20,21], limiting its ability to measure numerous gait metrics that are important for pre-surgical risk assessments, including step count, gait cadence, maximal oxygen consumption, and metabolic equivalents [10,22]. Marker-based methods have been combined with the Kinect to attempt to resolve this issue, but marker-based systems cannot be used in many clinical scenarios because they are cumbersome and require participants to wear colored markers, or some other wearable device, on multiple joints.

**Fig 1:**
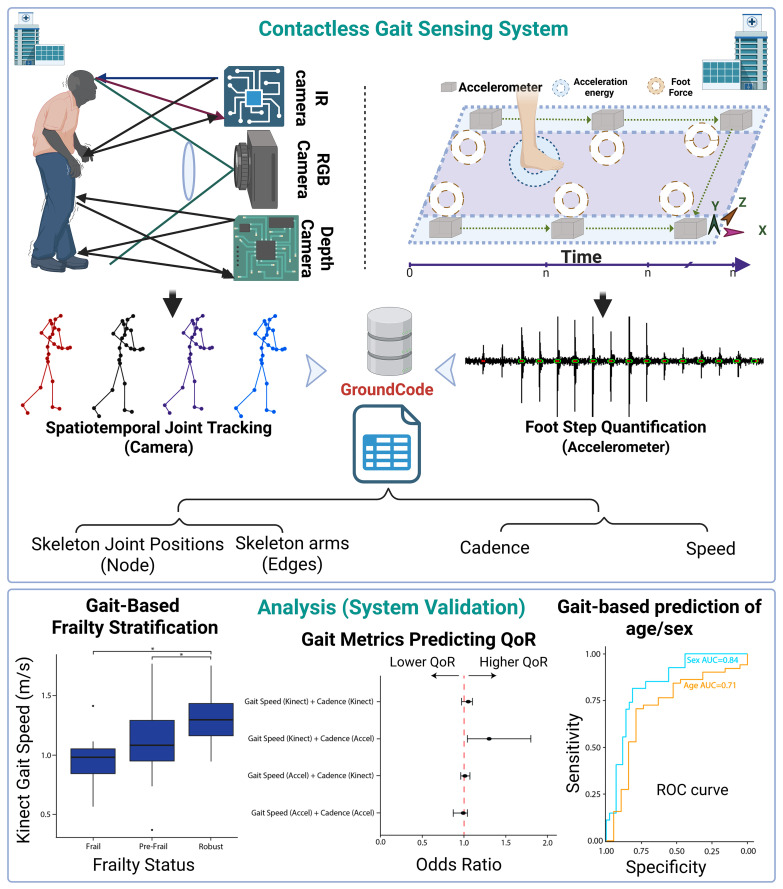
Contactless Gait Sensing System Overview. GroundCode integrates camera and accelerometer data to track joint movements and quantify footstep metrics. It provides dynamic gait tracking and personalized biometrics. This report integrates GroundCode within a surgical workflow, validates it against clinical gait metrics, and shows that pre-surgical gait features are associated with age, sex, frailty, and postoperative quality of recovery.

In this study, we report the development and validation of a low footprint, rapidly-deployable, entirely contactless sensing system for gait analysis in non-laboratory settings, called GroundCode (Fig 1). We designed this system to be fully integrated within a surgical workflow to address the known challenges of clinical implementation and integration faced by other technologies. GroundCode combines the Kinect with seven floor-mounted single-axis accelerometers, incorporating the robust spatiotemporal joint measurements of the Kinect with high-fidelity footstep detection and quantification of floor accelerometers. This multi-modal system addresses the Kinect’s limitations while maintaining a completely contactless sensor system. Using a prospective cohort study design (N = 77), we test the hypothesis that GroundCode can accurately measure gait speed and cadence by leveraging pelvis position from the Kinect and heel strike timing from the floor-mounted accelerometers. With our focus on clinical implementation, we compare GroundCode’s gait speed and gait cadence to clinical gait assessments relevant for surgery – stopwatch-derived gait speed and six-minute walk test distance. We validate that GroundCode-derived gait measurements can predict age and sex of participants, two characteristics that contribute to surgical risk and are known to have distinctive gait features. Lastly, we investigate if GroundCode-derived gait characteristics are associated with preoperative frailty (a well-known surgical risk factor) and postoperative quality of recovery.

## Methods

### Study population

**Fig 2:**
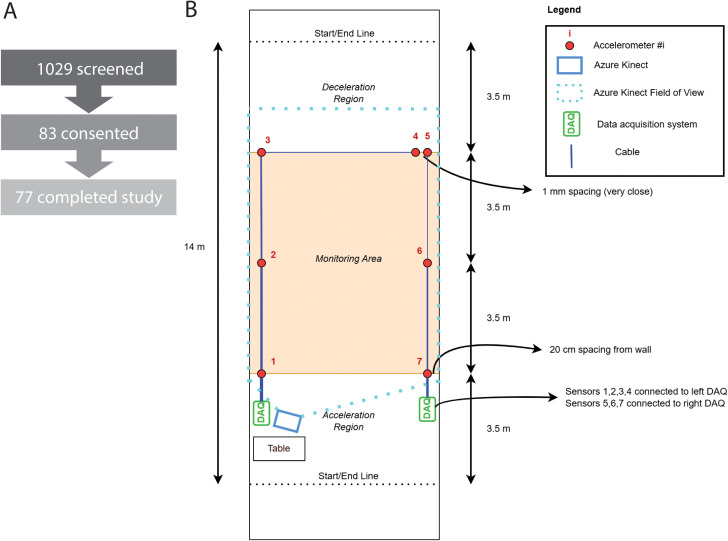
Specifications of cohort recruitment and GroundCode. (A) Screening and enrollment statistics for our cohort. (B) The design of the multi-modal contactless sensing system includes six single-axis floor accelerometers and one Kinect positioned at one end of the hallway. The Kinect field of vision includes the entire area monitored by the six accelerometers.

The study was approved by the Weill Cornell Medicine Institutional Review Board (IRB# 21–01023137). Adult patients scheduled for general surgery at Weill Cornell were considered eligible for study enrollment. Patients were excluded if they required walking assistance or had a contraindication for walking down a hallway or performing a six minute walk test, including bilateral blindness, hemiplegia, unstable angina, or oxygen dependence. The research team screened patients between March 3, 2022 and June 18, 2024, and reached out to patients by telephone to obtain informed consent prior to their procedure date. In total, 1029 patients were screened, 83 patients were consented, and 77 patients completed the study (Fig 2A). All walking trials were performed in an isolated 14-meter hallway on the morning of surgery in a clinical, non-laboratory setting. GroundCode was not permanently installed in the hallway, but instead was deployed and broken down for each patient trial.

### Design and deployment of the contactless sensing system

GroundCode is a system that integrates two contactless sensors, a Microsoft Azure Kinect DK (“Kinect”) and a grid of seven floor-mounted, single-axis accelerometers (PCB 393B04, numbered 1 through 7, Fig 2B) which measures the vertical vibration of the floor. The system was designed to be quickly deployed in a long hallway. The Kinect was placed at one end of the hallway and connected directly to a local laptop. Kinect data was processed in MATLAB using the open-source KinZ-Matlab toolbox which facilitates communication with the built-in Body Tracking SDK of 32 joint positions using the depth and video cameras. Our data acquisition script queries and stores these joint positions at approximately 30 frames per second. The accelerometer data was captured using two wireless data acquisition units (National Instruments cDAQ-9191) and transmitted over a local WiFi network to the laptop. Six of the seven accelerometers were placed in a 2x3 grid with 5.0m x 3.5m spacing and were used for monitoring and for providing redundancy in case of sensor failure or low signal to noise ratio. Accelerometers 1, 2, and 3 were placed on the left side of the hallway and connected to a data acquisition unit (DAQ) on the left side of the hallway (Fig 2B). Accelerometers 5, 6, and 7 were placed on the right side of the hallway and connected to a DAQ on the right side of the hallway (Fig 2B). The remaining accelerometer (#4 in Fig 2B) was placed immediately next to accelerometer #5 on the right side of the hallway, but connected to the left DAQ, as a way to time-synchronize between the two wireless DAQs at the post processing stage (described below in “Quantification of gait metrics with footstep data”). A wireless system was chosen in order to reduce cabling, but a fully cabled system could have been used to avoid the time-synchronization step. The data was processed in MATLAB using the National Instruments NI-DAQmx driver and a custom-built function library. The accelerometers were each sampled at 16,384 samples per second and the raw acceleration data was saved for further processing. Automated collection of the Kinect and accelerometer data began simultaneously when a participant walked through the hallway. In addition, start and stop times were manually recorded when each participant crossed the starting and finishing lines to manually calculate gait speed (referred to as “stopwatch time”).

### Quantification of gait metrics with body tracking

#### Gait speed.

To estimate gait speed, the pelvis position was continuously captured by the Kinect, and the pelvis position defined at each time i as $\left(x_{i}^{pelvis},\;y_{i}^{\;pelvis},\;z_{i}^{pelvis}\right)$. The distance travelled by the pelvis joint at time i was defined as:


$$d_{i}^{pelvis}=\sqrt{{\left(x_{i}^{pelvis}-x_{\text{0}}^{pelvis}\right)}^{\text{2}}+{\left(y_{i}^{\;pelvis}-y_{\text{0}}^{\;pelvis}\right)}^{\text{2}}+{\left(z_{i}^{pelvis}-z_{\text{0}}^{pelvis}\right)}^{\text{2}}}$$

where the point $\left(x_{\text{0}}^{pelvis},\;y_{\text{0}}^{\;pelvis},\;z_{\text{0}}^{pelvis}\right)$ was the position of the pelvis when it was first detected by the body tracking algorithm. The raw distance time series is then automatically processed to remove segments with data drop-outs, usually at the beginning and end of each trial. Data drop-outs of 2 samples or less were interpolated based on adjacent samples. Next, each subject’s gait speed was estimated as the slope of a least squares linear fit of the three-dimensional distance of the pelvis joint over time. The least squares regression was defined as:


$$d_{i}^{pelvis}=gs*t_{i}+d_{\text{0},}^{pelvis}\;$$

where gs represents the gait speed (slope of the distance) and $d_{\text{0}}^{pelvis}$ represents the initial distance.

Due to the fact that the data could include periods of acceleration/deceleration that would occur outside of the monitoring area, we implemented an automated process to identify only the portion of the pelvis position that was changing linearly. The data was trimmed iteratively (by removing data points from both ends) until the R^2^ coefficient of the linear fit exceeded 0.98. In about 10% of cases, the data was manually trimmed to improve the quality of the fit.

#### Gait cadence.

To estimate gait cadence, we first created a “foot swing” signal, defined as the relative motion between each ankle joint and the pelvis joint,


$${FS}_{i}=\sqrt{{\left(x_{i}^{ankle}-x_{i}^{pelvis}\right)}^{\text{2}}+{\left(y_{i}^{ankle}-y_{i}^{\;pelvis}\right)}^{\text{2}}+{\left(z_{i}^{ankle}-z_{i}^{pelvis}\right)}^{\text{2}}}$$

where $\left(x_{i}^{ankle},\;y_{i}^{ankle},\;z_{i}^{ankle}\right)$ is the position of one of the ankle joints (left or right) at time i. The gait cadence was estimated by taking the Fast Fourier Transform of both FS signals (left and right foot), then finding the average of the maximum frequency (Hz) in each signal, $f_{max}^{avg}.$ The cadence (steps per minute) was calculated as


$$cad=\text{1}\text{2}\text{0}\;f_{max}^{\;avg}$$

### Quantification of gait metrics with footstep vibration data

Both gait speed and gait cadence were estimated from footsteps identified by seven floor-mounted accelerometers. The raw accelerometer data was first linearly detrended to remove any zero offsets. Then it was forward-backward filtered using a high-pass filter at 5 Hz (Butterworth IIR, second order) to remove low-frequency drift. Finally, it was forward-backward filtered using a low-pass filter at 250 Hz (Butterworth IIR, second order) to remove high-frequency mechanical noise. After the raw data was processed, the accelerometers were time synchronized by taking the time lag of the maximum cross correlation between sensors 4 and 5 (which were placed immediately adjacent to each other side-by-side, Fig 2B), and then shifting the time series for sensors 5, 6, and 7 appropriately (Fig 2B). After synchronization, sensor 4 was discarded from the analysis as its data was nearly identical to sensor 5.

Data was then aggregated from all sensors into a single signal, called Maximum Signal to Noise Ratio (${SNR}_{max}$), using the following procedure. First, the average signal power (acceleration squared) for each sensor was calculated using a moving window of 0.02 seconds with no overlap. Next, the average signal power for each sensor was normalized by the median power for that sensor to produce an SNR function for each sensor. Third, the ${SNR}_{max}$ was computed by taking the maximum SNR across all sensors for each window. Peaks in the ${SNR}_{max}$ were detected using a peak-finding algorithm (MATLAB *findpeaks* function) with the following criteria:

1. Minimum Peak Height =  median(${SNR}_{max}$) + 9*$\sigma_{SNR}$, where $\sigma_{SNR}\;$is the standard deviation of the ${SNR}_{max}$ signal after removal of points above 3 median absolute deviations (to exclude peaks from the calculation).
2. Minimum Peak Distance = 0.3 seconds

#### Gait speed.

Gait speed was estimated using an energy-based localization approach as previously described [23]. Briefly, the method first estimates the position of each footstep based on the relative energies of the raw acceleration time series of each sensor for each detected step, assuming an exponential decay in energy from the footstep location. For improved robustness to poor instances of localization, the method updates the detected steps using Kalman filter with a constant speed kinematic model. This corrects for foot step locations which may be mistakenly identified far from the previous one. Because the hallway floor is not a homogeneous medium, the exponential energy decay is a poor idealization which can lead to noisy footstep location estimates.

#### Gait cadence.

To calculate cadence from ${SNR}_{max}$peaks, the list of time differences in seconds (Δt) between consecutive peaks was computed, which represent step times. The values of Δt are distributed around the mean step time when cadence is constant and step capture is accurate. Missed steps or pauses in walking will appear as outliers in the distribution. After removing outliers, we computed the cadence in steps per minute as 60/mean(Δt).

### Clinical data collection and assessment

All consented patients had their age, sex, and medical comorbidities documented. Each patient performed a walking trial in plain clothes which consisted of walking 14m between two clearly marked lines. The initial 3.5m and final 3.5m served as acceleration and deceleration regions, respectively. The middle 7m of the walking space (orange area, Fig 2B) provide an estimate of steady-state walking. Patients typically performed four trials, two at a regular pace and two at a fast pace. Because of time constraints regarding clinical care, some participants did not complete all four trials. Research personnel prompted each patient to begin their trial and manually recorded start and stop time to derive stopwatch-derived gait speed.

A subset of patients were taken to an adjacent hallway to perform a six minute walk test. For the six-minute walk test, a 20m track was marked with floor tape. The patient was prompted to begin the test and walk from one end of the track to the other. After the conclusion of the test, total distance was recorded in feet.

### Calculating test-retest reliability

Test-retest reliability was performed for regular and fast walking separately for individuals who completed repeated trials. Intraclass correlation coefficients were calculated for gait speed and gait cadence during regular and fast walking using the irr package in R. Permutation testing was performed by randomly shuffling patient labels 10,000 times and recalculating intraclass correlation coefficients each time. True intraclass correlation coefficients were then compared to the permuted distribution to determine the false discovery rate (Q).

### Gait speed and cadence validation

GroundCode-derived gait speed (Kinect) was compared to stopwatch-derived gait speed at regular and fast walking speeds independently. Gait speed measurements for individuals across two trials were averaged. In the regular walking cohort, five participants had average stopwatch-derived gait speed > 2.5 m/s due to user stopwatch error, so they were excluded. For both regular and fast walking, outliers also were empirically determined by calculating the difference between GroundCode- and stopwatch-derived gait speeds and investigating the distribution. Because they likely represented device malfunction or stopwatch-user error, trials with calculated differences greater than 2.5 standard deviations from the mean were excluded. Pearson correlation was calculated between stopwatch-derived and device-derived gait speed at regular and fast walking speeds and *r* and p-values were reported.

To determine the quality of the accelerometer detection, we investigated the distribution of instantaneous cadence to ensure that steps with significant decreases in instantaneous cadence were rare. Missed steps result in artificially increased amounts of time between detected steps, leading to precipitous declines in instantaneous cadence. For each walking trial, instantaneous gait cadence was calculated for each step and Z-normalized by the trial mean and standard deviation. The distribution of Z-normalized instantaneous gait cadence was plotted for all steps across all trials, and colored by whether the step was detected or missed. We also compared steps detected by the accelerometer and filtering algorithm with total number of steps taken by each patient. Total number of steps was determined from the accelerometer data by adding detected steps to missed steps. Pearson correlation was calculated and *r* and p-values reported. Lastly, GroundCode-derived gait cadence (accelerometers) at regular walking speed was compared to six minute walk test performance. Pearson correlation was calculated between gait cadence and distance traveled during the six-minute walk test, and *r* and p-values were reported.

### Estimating the relationship between gait metrics, preoperative surgical risk, and postoperative outcomes

Age differences in gait speed and cadence were investigated by calculating Pearson correlation between age and gait metrics. Mann Whitney U was calculated to compare gait metrics between participants greater than or less than 60 years. To distinguish male and female patients, body area was estimated by multiplying shoulder width by body length. Body length was derived from Microsoft Kinect by adding torso length to the average length of right and left legs. Body area for the entire cohort was normalized by the mean and standard deviation of female body area. Mann Whitney U was calculated to compare normalized body area between men and women. To estimate GroundCode prediction accuracy for age and sex groups, leave-one-out cross validation was performed. Briefly, each sample was iteratively withheld as the testing sample with the remaining samples used as training data for model building. For each iteration, logistic regression models were calculated on the training data for sex and age respectively. For sex-based models, sex was the dependent variable and body area was the independent variable. For age-based models, binarized age group was the dependent variable and gait speed was the independent variable. The testing sample was predicted based upon the logistic regression model of the training data and overall model accuracy was calculated by averaging the accuracy of each model in the leave-one-out cross validation process.

Pre-surgical frailty was measured on the same day as the walking trial for each patient. The Fried Frailty Index was used to quantify frailty [24]. Robust, prefrail, and frail patients were identified based upon standard Fried Frailty criteria (Robust = 0, Prefrail = 1–2, Frail>2, respectively). Average gait speed was compared between the three groups by t-test. Post-surgical recovery was assessed in this same cohort on the first day after surgery using the Quality of Recovery-15 (QoR-15) questionnaire, which includes self-reported assessments of pain, sleep, and fatigue [25]. Outcomes were binarized. Patients with QoR-15 scores > 121 were classified as good recovery and patients with QoR-15 scores <= 121 were classified as poor recovery. This binarization was based upon previously validated cut offs [26]. Association between gait speed-cadence interactions and QoR-15 status was estimated with logistic regression.

## Results

### Body tracking and footstep metrics are quantifiable after rapid deployment in non-laboratory setting

**Fig 3:**
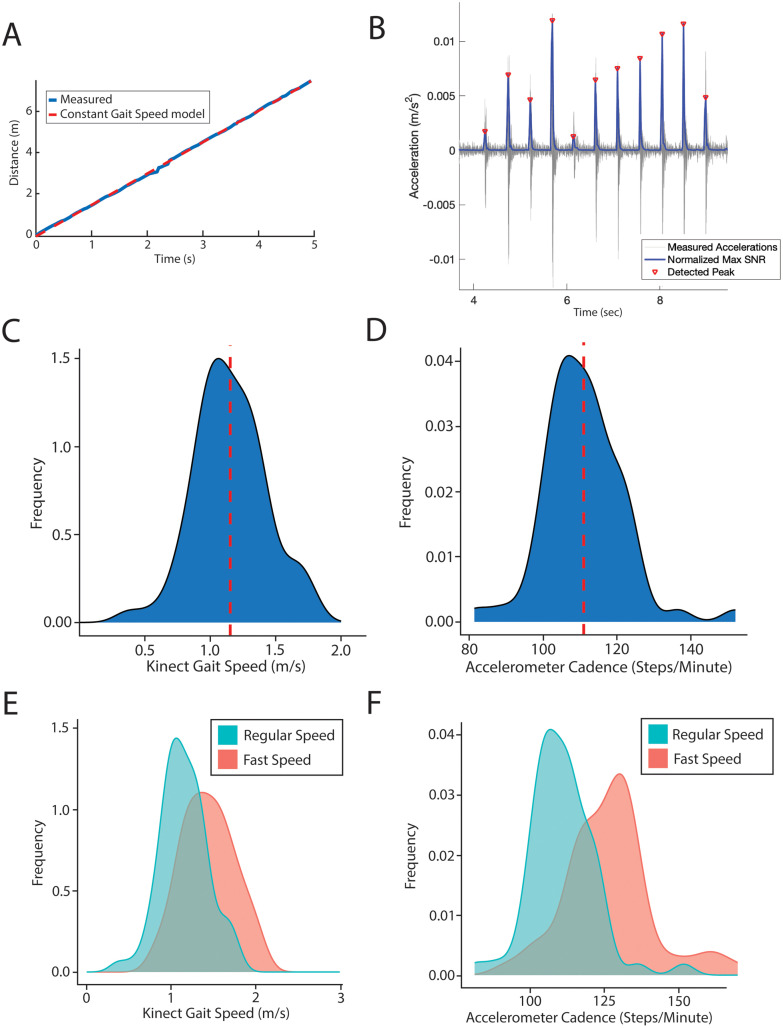
Measuring gait speed and cadence and their distributions. (A) Kinect-based gait speed measurement derived from pelvic location over time. (B) Accelerometer-based footstep detection with red dots denoting detected footsteps and single-axis acceleration represented along the y-axis. (C,D) Distribution of gait speed and cadence measurements in the cohort when walking at regular pace. Red dotted lines reflect the means (1.2 m/s and 111 steps/minute, respectively). (E,F) The distributions of gait speed (E) and cadence (F) during regular (blue) and fast (red) walking.

GroundCode integrates data from a single Microsoft Kinect and seven single-axis floor accelerometers (Fig 1) and was successfully deployed for each participant of the cohort in a clinical, non-laboratory setting (N = 77, Fig 2). The average age of the cohort was 66 years (standard deviation = 13), and 60% of the cohort was male (Table 1). As each participant walked through the designated hallway, GroundCode automatically quantified numerous skeletal and footstep metrics. Thirty-two joints were captured from the Microsoft Kinect component, and pelvis position was reliably estimated over time to derive gait speed (Fig 3A). The floor-mounted accelerometers detected footsteps (Fig 3B) from which derivative features like gait cadence (steps per minute) were estimated (See Methods). In our prospective cohort, participants performed two trials at regular pace and two trials at fast pace. Gait speed varied from 0.4 m/s to 1.8 m/s (mean = 1.2m/s, sd = 0.27) and gait cadence varied from 81 steps/minute to 152 steps/minute (mean = 111 steps/minute, sd = 11) when participants walked at a regular pace (Fig 3C, 3D). Participants performing fast walking trials had significantly greater Kinect-derived gait speeds (Kolmogorov-Smirnov test, D = 0.4, p = 2.8x10^-4^) and accelerometer-derived cadences (Kolmogorov-Smirnov test, D = 0.6, p = 1x10^-9^) than when they performed trials at a regular pace, increasing their gait speed by 25% and their cadence by 14% (Fig 3E, 3F). These analyses provide internal validation of measurement sensitivity.

**Table 1:** The cohort’s general characteristics, comorbidities, and physical activity frequency is reported. Mean, median, standard deviation (SD), and interquartile ranges (IQRs) are noted and labeled as appropriate. For comorbidities, total number of patients in our cohort diagnosed with each disease is reported (n), as is the percentage (%).

General Characteristics	
Age (Median, IQR, Mean, SD)	68, (46.5-89.5), 66, 13
Sex (% Female, % Male)	40%, 60%
Race (%Asian, %Black, %Hispanic, %White, %Unreported)	4%, 12%, 1%, 71%, 12%
BMI (Median, IQR, Mean, SD)	28.6, (20.5-36.7), 28.8, 6.6
Smoking (%Current, %Past, %Never)	1%, 44%, 55%
Dyspnea (% no SOB, %SOB with moderate exertion)	86%, 14%
Current Alcohol consumption (% Yes, % No)	50%, 50%
**Comorbidities**	
Atrial Fibrillation (n, %)	9, 12%
Arthritis (n, %)	15, 20%
Coronary Artery Disease (n, %)	13, 17%
Peripheral Artery Disease (n, %)	4, 5%
History of Stroke/Cerebrovascular Accident (n, %)	8, 11%
History of Myocardial Infarction (n, %)	4, 5%
Respiratory Disease (n, %)	13, 17%
Diabetes (n, %)	20, 26%
Depression (n, %)	13, 17%
Anxiety (n, %)	10, 13%
**Frequency of Moderate Physical Activity**	
>3 times per week (n, %)	33, 43%
1-2 times per week (n, %)	14, 19%
1-3 times per month (n, %)	6, 8%
Hardly Ever (n, %)	23, 30%

### Test-retest reliability of gait speed and cadence at regular and fast walking speeds

**Fig 4:**
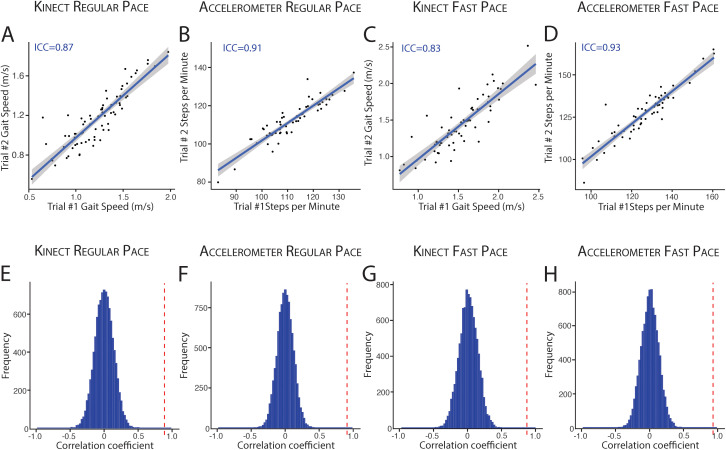
Test-retest reliability of gait speed and cadence during regular and fast pace walking. (A-D) Intra-class correlation for gait speed and steps/minute across repeated trials (Trial #1 and Trial #2) at both regular and fast walking pace. (E-H) Permutation-based correlation coefficients (blue histogram) for devices and walking speeds corresponding to the row above (A-D). True intra-class correlation coefficients are denoted by dotted red lines. ICC = intra-class correlation.

For patients who participated in repeated walking trials (“Trial 1” and “Trial 2”), we calculated intra-class correlation coefficients (ICC) between trials to determine test-retest reliability of gait speed (Kinect) and cadence (single-axis accelerometers) at both regular (N = 71) and fast (N = 62) walking speeds. Intra-class correlation was strong for both gait speed (ICC = 0.87, P = 1.6x10^-15^) and cadence (ICC = 0.91, P = 8.6x10^-25^) at regular walking speeds (Fig 4A, 4B). With fast walking, intra-class correlation coefficients for gait speed (ICC = 0.83, P = 2.6x10^-18^) and cadence (ICC = 0.93, P = 1.1x10^-27^) remained robust (Fig 4C, 4D). Cadence and speed estimates during regular and fast walking remained reliable across patients who self-report frequent energetic physical activity (“>3 times per week”; Cadence ICC = 0.84, P < 0.05, Speed ICC = 0.91, P < 0.05) as well as patients who are more sedentary (“Hardly ever/never”, Cadence ICC = 0.95, P < 0.05; Speed ICC = 0.81, P < 0.05) (S1 Fig). To confirm that high intra-individual correlation was not biased by low variance in speed and cadence across the population, permutation testing was performed (N = 10,000). Intra-individual correlation is substantially greater than correlation coefficients based upon permuted pairs of individuals (Fig 4E–4H, Q < 0.0001, for both speed and cadence).

### Kinect and accelerometers show complementary strengths in measurement reliability

To demonstrate the complementary strengths of the Kinect and single-axis accelerometers, we compared gait speed and cadence measured by each device. In Trial #1 and Trial #2 at regular walking pace, the intra-class correlation of gait speed was significantly greater from the Kinect (ICC = 0.87) than the accelerometers (ICC = 0.53) (S2 Fig). Likewise, gait cadence was more reliably measured by the accelerometer (ICC = 0.91) than the Kinect (ICC = 0.1) (S2 Fig). These results were also confirmed in fast walking trials, demonstrating that the Kinect more reliably estimates speed while the accelerometers more reliably estimate cadence.

### Validating contactless gait metrics with stopwatch time and six-minute walk test

**Fig 5:**
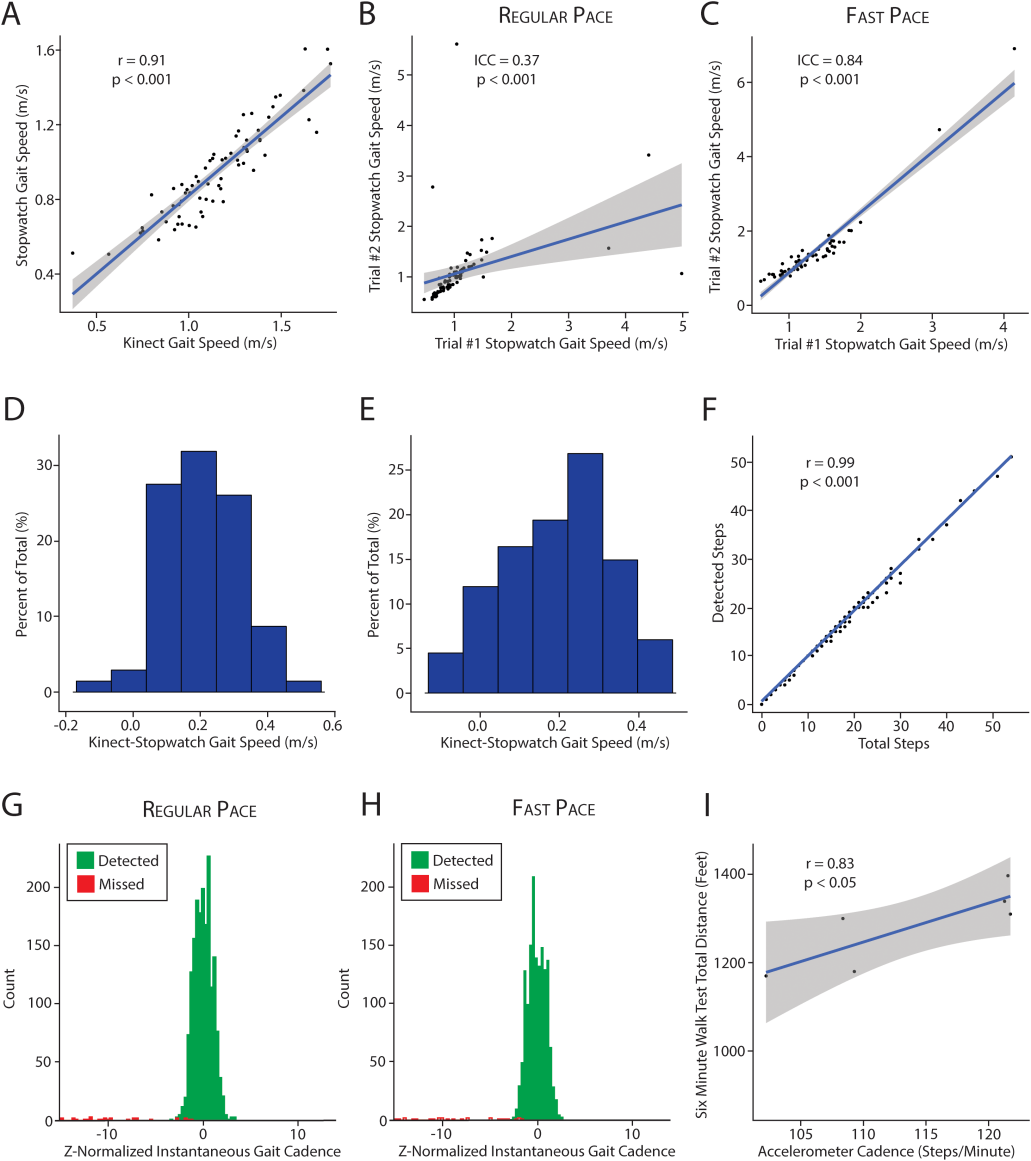
Comparing contactless gait metrics with clinical gait assessments. (A) Correlation between Kinect-derived vs. stopwatch-derived gait speed (p < 0.05). (B,C) Intraclass correlation coefficient for stopwatch gait speed at regular and fast pace, respectively. Two trials were performed at regular pace and two trials were performed at fast pace. The distribution of the difference between Kinect and stopwatch derived gait speed during regular (D) and fast (E) pace walking. Values > 0 reflect faster Kinect-based gait speeds. (F) Comparing total steps with number of detected steps by the accelerometer. (G,H) The distribution of Z-normalized instantaneous cadence for all steps taken by all patients in the cohort. Detected steps are in green. Missed steps are in red. Missed steps demonstrate a significant decrease in instantaneous gait cadence because the time between detected steps is artificially increased. These missed steps (red) are very rare at both regular (G) and fast (H) pace. (I) Accelerometer based cadence vs six minute walk test distance (p < 0.05).

Next, we validated gait metrics against standard clinical gait assessments, stopwatch-based gait speed and six minute walk test. Device-derived gait speed (Kinect) is strongly correlated with stopwatch-derived gait speed during regular walking (N = 69, r = 0.91, P = 8x10^-27^) (Fig 5A). This remained robust for fast walking (N = 67, r = 0.92, P = 5x10^-28^). Notably, the intra-individual correlation for device-derived gait speed (N = 71, ICC = 0.87, P = 1.6x10^-15^, Fig 4A) was significantly greater than stopwatch-derived gait speed during regular walking (N = 71, ICC = 0.37, p < 6x10^-4^, Fig 5B). During fast walking, the intra-individual correlation for gait speed was similar between the stopwatch (N = 61, ICC = 0.84, p = 4x10^-18^, Fig 5C) and device (N = 62, ICC = 0.83, P = 2.6x10^-18^, Fig 4C).

Because of the poorer intra-individual correlation for stopwatch times during regular walking, we investigated the deviation between stopwatch and device-derived gait speed. Participants had lower gait speeds when measured with a stopwatch. The median difference between device- and stopwatch-derived gait speed was 0.19m/s at regular pace (median absolute deviation = 0.1) and 0.2m/s at fast pace (median absolute deviation = 0.1) (Fig 5D, 5E).

To validate gait cadence, we compared the number of steps detected by the single-axis accelerometers to the overall steps taken by each participant and found that they were highly correlated (r = 0.99, p < 1x10^-300^) (Fig 5F). In 72% of trials, all steps were detected. In 96% of trials, only 2 or fewer steps were missed. As a consequence, the instantaneous gait cadence estimate for each step was highly robust, with only rare instances of missed steps causing biased (extremely low) estimates of instantaneous gait cadence at regular (Fig 5G) and fast pace (Fig 5H). Lastly, we examined gait cadence in a subset of patients who also performed the six-minute walk test, another clinical functional assessment associated with surgical risk. We confirmed that device-derived cadence (steps/minute) was well correlated with total distance walked in the six-minute walk test (N = 6, r = 0.83, P < 0.05, Fig 5I).

Lastly, additional analyses showed that gait speed from the accelerometer is not associated with stopwatch-based gait speed (p > 0.05, S2 Fig). Likewise, cadence estimated from the Kinect is not associated with the six minute walk test (p > 0.05, S2 Fig). Together, this supports that each component of GroundCode is associated to relevant clinical gait assessments and provides complementary information to capture a greater number of gait features reliably.

### Predicting age and sex with body tracking and footstep metrics

**Fig 6:**
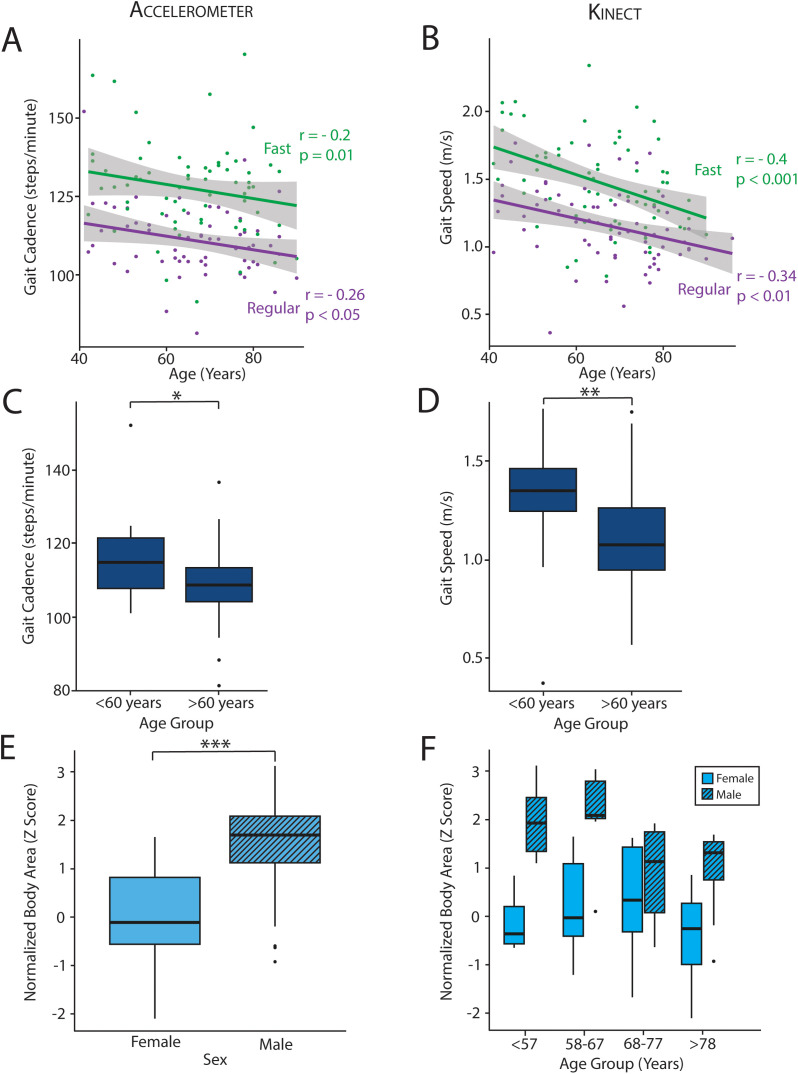
GroundCode identifies age and sex differences. Gait cadence (A) and gait speed (B) decrease with age, irrespective of fast paced (green) or regular paced walking (purple). (C,D) Gait cadence (p = 0.02) and speed (p = 1.2x10^-3^) distinguishes people older than 60 years old from those younger than 60 years old. (E) Men have a greater Kinect-derived body area than women (p = 4.9 x10-7). (F) This sex difference is consistent across all age quartiles in our cohort (age bracket quartiles denoted on x axis of F). * p < 0.05, ** p < 0.01, ***p < 0.001.

**Fig 7:**
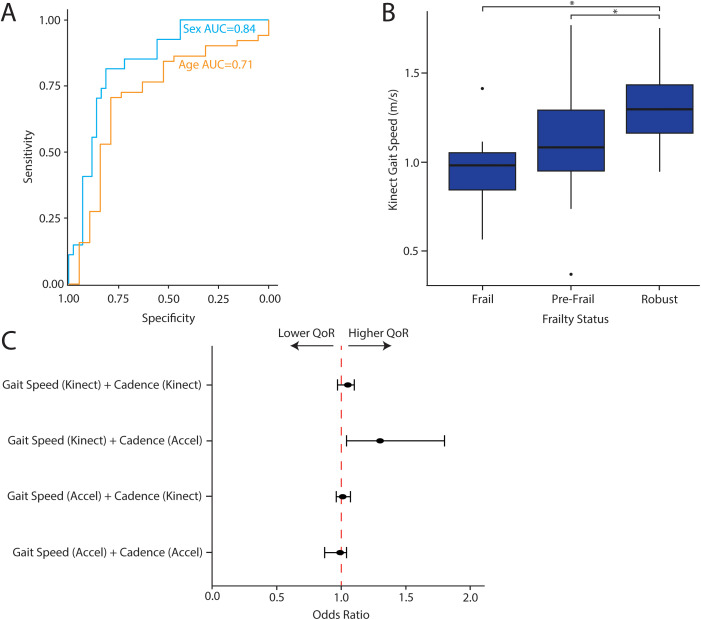
Associating contactless gait metrics with preoperative risk and postoperative outcomes. (A) Leave-one-out cross validation receiver operating curves for predicting age and sex from Kinect-derived gait speed and body area, respectively. (B) Kinect-derived gait speed (m/s) compared between robust, pre-frail, and frail patients, defined by Fried Frailty Index. Frail and pre-frail patients have significantly slower gait speed than robust patients (*p < 0.05). (C) Odds ratio and 95% confidence intervals associating quality of recovery (measured by QoR-15) to gait speed and cadence. The odds ratio from the speed*cadence interaction term is plotted from four different models that incorporate gait speed and cadence from different combinations of devices (noted in y-axis). Interaction between Kinect-derived gait and accelerometer-derived cadence is associated with better quality of recovery (p < 0.05).

Since gait has well-characterized age and sex-specific features [27,28], we hypothesized that GroundCode could distinguish age and sex based upon body tracking and footstep metrics alone. Importantly, various surgical outcomes also vary by both age and sex, making age/sex predictions relevant for the development of gait-based surgical risk assessments [29,30]. We found that age was negatively correlated with both cadence (N = 64, r = -0.26, p = 0.03) and gait speed (N = 64, r = -0.34,p = 3.5x10^-3^) when walking at a regular pace, corroborating previous studies (Fig 6A, 6B). Individuals who met the World Health Organization threshold for old age (>60 years old) had lower cadence (Mann-Whitney U, P = 0.02) and gait speed (Mann-Whitney U, 1.2x10^-3^) compared to younger participants (Fig 6C, 6D). During fast walking, age remained correlated with cadence (N = 66, r = -0.2, p = 0.1) and speed (N = 66, r = -0.4, p = 4x10^-4^). In addition to distinguishing age, we also investigated whether device-derived body area estimates distinguish men and women. We found that men had a device-derived body area approximately 1.5 standard deviations greater than the average woman in our cohort (N = 70, W = 129, 4.9 x10^-7^) (Fig 6E). This difference was similar across most age quartiles in our cohort (Fig 6F). To estimate how well age and sex can be predicted from single features (gait speed and body area, respectively), leave-one-out cross validation was performed and demonstrated good performance (AUC = 84%, and 71%, respectively) (Fig 7A). Together, these analyses corroborate previous literature findings regarding age and sex and provide further validation of GroundCode’s reliability when deployed by non-technical personnel in a real-world clinical environment.

### Gait metrics are associated with frailty before surgery and quality of recovery after surgery

We investigated if gait metrics are associated with surgical risk and outcomes. We investigated comorbidities for which at least 10 patients in our cohort had a positive diagnosis and found no differences in gait speed and cadence (Table 2). Next, we investigated frailty since it increases the risk of poor surgical outcomes and is commonly assessed before surgery [31]. We measured preoperative Fried Frailty Index in our cohort and found that gait speed is correlated with Fried Frailty Index. The average gait speed for robust patients was 1.3 m/s, while frail and prefrail patients was 0.99 and 1.18 respectively (p < 0.05, Fig 7B). Next, we hypothesized that preoperative gait metrics are associated with postoperative recovery from surgery. We assessed surgical recovery with the Quality of Recovery-15 (QoR-15) questionnaire on postoperative day 1, which is a robust and reproducible assessment of patient recovery and a predictor of postoperative complications [25,32]. We found that the interaction between faster gait speed (Kinect) and greater gait cadence (accelerometer) before surgery is associated with better recovery after surgery (interaction term, p < 0.05, Fig 7C). Notably, models with gait speed and cadence measured from the same device, as well as models with accelerometer-based gait speed and Kinect-based cadence (Fig 7C), are not associated with quality of recovery after surgery.

**Table 2:** Average speed and cadence for people with (+) and without (-) each comorbidity is reported. T-test p values comparing speed and cadence between people with and without each disease are also reported.

Comorbidity	Speed, m/s (+)	Speed, m/s (-)	Speed P-value	Cadence, steps/min (+)	Cadence, steps/min (-)	Cadence P-value
Arthritis	1.1	1.2	0.42	110	111	0.68
Anxiety	1.2	1.2	0.87	108	111	0.22
Respiratory Disease	1.02	1.2	0.15	108	111	0.25
Diabetes	1.1	1.2	0.5	115	110	0.21
Depression	1.1	1.2	0.2	110	111	0.8
Coronary Artery Disease	1.1	1.2	0.2	110	111	0.8

## Discussion

Digital health technologies need to be integrated within clinical workflows in order to maximize their potential benefits. To best take advantage of the significant research efforts that continuously create better technologies, equally careful consideration must be given to address implementation challenges. In this paper, we describe an ambient intelligence system that is fully integrated within the surgical workflow and built into the infrastructure of a hospital space used for preoperative evaluation. Though its monitoring space is limited to a single hallway (unlike wearables), it has the great advantage of having a straightforward pathway to clinical adoption. Importantly, we provide evidence that it reproducibly quantifies gait features on-par with the clinical gold standards used in preoperative functional assessments, validates previously described age and sex differences in gait, and is correlated to preoperative frailty and postoperative recovery. This is achieved despite its limited monitoring space and its deployment outside of a laboratory by non-technical personnel.

Contactless sensors create new opportunities to design more effective and equitable systems for collecting and analyzing biometric healthcare data. GroundCode was specifically designed with multi-sensor integration in mind, so that the weaknesses of each sensor are mitigated by strengths of the other. For instance, well-known limitations in the Kinect’s ability to track ankle position make footstep detection and cadence challenging to estimate, while the single-axis floor accelerometer makes highly robust cadence estimates with excellent footstep detection. Self-occlusions, camera resolution, and clothing variations, like hospital gowns, can contribute to the noise in Kinect-estimated ankle and foot positions, but a complementary technology like single-axis accelerometers can make up for these limitations. Because of these challenges, the Kinect makes poor estimates of gait cadence and the 6-minute walk test, both of which are clinically used in surgical risk stratification as proxies for cardiorespiratory fitness, functional status, and postoperative outcomes. Complementing the Kinect with single-axis accelerometers allowed us to capture cadence and 6-minute walk test with good accuracy. These measurements can be used to identify patients with increased perioperative risk. In addition, we show that the interaction between accelerometer-derived cadence and Kinect-derived speed before surgery was not only associated with preoperative risk, but also the quality of recovery after surgery. This is further support for the utility in integrating accelerometers (and cadence estimates) with the Kinect.

Using complementary technologies has additional benefits. For instance, camera technologies introduce elevated privacy concerns in clinical situations, so having alternative modalities can provide redundancy in scenarios where a Kinect cannot be used. The current report focuses on the potential advantages of gait analysis with the dual technologies of GroundCode, but future work will also explore integrating this system with a network of Kinects, which could reduce the effects of self-occlusions though not fully mitigate the difficulties in cadence estimates. GroundCode can also be integrated with other contactless and wearable technologies, like heart rate monitors, chemical sensors, and activity trackers. Because of its ability to interface with other technologies, it can also be incorporated in efforts to build smart homes and digital clinics to facilitate healthy aging and aging in place [33].

This proof-of-principle study evaluated the reliability and validity of GroundCode-derived estimates of gait speed and cadence and demonstrated their association with preoperative risk (age, sex, and frailty) and postoperative quality of recovery. Numerous gait features, including gait speed and cadence, are clinical predictors of functional status, hospitalization risk, cognitive decline, and overall health [1,2,34–36], and GroundCode may improve the specificity of gait-related clinical classifications that incorporate speed and cadence. For example, in our study, thirty percent (30%) of the cohort had a stopwatch-derived gait speed of <0.8m/s, a threshold for predicting mortality and poor clinical outcomes [2], but GroundCode-derived gait speed only corroborated 25% of these cases. Taken together, this evidence suggests GroundCode may have significant utility in longitudinal monitoring and in early disease detection while placing no burden on the individual to maintain, charge, or wear a device. Since GroundCode is rapidly deployable and does not require specialized laboratory equipment or personnel to operate, it may be a feasible option for various health-monitoring strategies outside of the hospital, like home-based frailty assessments and digital preoperative clinics. These possible future applications motivate further study of contactless systems in various clinical and non-clinical environments.

There are several limitations to GroundCode that should be considered in future experiments and implementations. Our study was conducted in a single hallway but environmental variability may contribute to differences in data quality. Accelerometer-based gait detection works best when participants are barefoot on hard floors, and additional cushioning between the heel and floor (like soft soles or floor carpet) will decrease the signal-to-noise ratio and increase the probability of missed steps. The patients in this study walked on a hard floor with shoes, generating reliable results. In environments with less desirable conditions, signal-to-noise ratio can be improved by increasing the sensor density on the floor, but it may pose challenges if a sparse system of accelerometers is desired, as we have described in this manuscript. With respect to the Kinect, lighting variations are a possible limitation. Very low lighting would affect Kinect’s body tracking performance, but normal variations in lighting are likely unproblematic since the Kinect is equipped with infrared camera which is robust to changes in lighting. In addition to limitations of individual devices, precise synchronization between Kinect and accelerometer systems is an additional challenge. Because gait speed and cadence estimates do not depend on synchronization between the two systems, this was not a concern for the current study, but would be a challenge for any study requiring precise synchronization.

In addition to technical limitations, it is important to acknowledge that GroundCode was compared to clinical gait metrics relevant to surgical risk due to the study’s emphasis on clinical integration. However, future studies can compare GroundCode’s accuracy to other laboratory and consumer technologies. Specialized sensing pads, like the GAITRite and the Zeno Walkway, or a multi-camera motion-capture system, like Vicon, offer standardized gait measurements that can be used to fine-tune and better calibrate GroundCode. Importantly, one component of GroundCode, the Kinect, has already been compared to Vicon, wearables, and pressure-sensitive walkways (including GAITRite), demonstrating comparable results for various body movement and gait metrics including gait speed [11,37–42]. This suggests that GroundCode will also perform comparably to these other gait systems. Furthermore, GroundCode integrates Kinect with single-axis floor accelerometers, but other vibration-based sensors have also been used to study gait and can also be investigated in future studies. For example, floor-mounted geophone vibration sensors convert ground motion velocity into voltage and reliably capture gait features, can detect gait abnormalities, and can identify clinically-relevant features of musculoskeletal diseases, like muscular dystrophies [43,44]. Single-axis accelerometers were chosen for this study because they are cost effective and have greater sensitivity to the high frequency changes characteristic of footsteps, however, a head-to-head comparison of accelerometers and geophones for gait detection and clinical prediction can provide value information for future ambient sensor designs.

## Supporting information

S1 Fig Test-retest reliability in patients with different baseline activity levels. Trial #1 and Trial #2 at regular walking pace showed high intra-class correlation coefficients for cadence and gait for patients with high physical activity (Panels A and C, self-report frequent energetic physical activity >3 times per week) as well as patients who have low physical activity (Panels B and D, self-report frequent energetic physical activity hardly ever/never). All intra-class correlation coefficient p values were < 0.05.
(TIF)

S2 Fig Kinect and Accelerometer metrics have complementary strengths. (A) Kinect-derived gait speed has higher intra-class correlation coefficients at both regular and fast pace than accelerometer-derived gait speed. (B) Accelerometer-derived cadence has higher intra-class correlation coefficients at both regular and fast pace than Kinect-derived cadence. Red dotted lines mark the thresholds for poor (< 0.5), moderate (0.5-0.75), and high (>0.75) intra-class correlation. (C) Accelerometer-derived gait speed is not associated with stopwatch-derived gait speed (p > 0.05). (D) Kinect-derived cadence is not associated with six minute walk test distance (p > 0.05).
(TIF)

S3 Fig The GroundCode system deployed in a non-laboratory setting. The GroundCode hallway with the Kinect in the foreground (connected to a laptop). The six accelerometer positions are marked by orange cones along the left and right sides of the hallway. The middle accelerometer position is marked by dual cones on both the left and right sides of the hallway. Accelerometers are connected to data acquisition units seen in the foreground. The beginning of the monitoring area is marked by the blue line.
(TIF)
