# Supplementary figures and images for: Development and validation of a multi-modal contactless sensing system for surgical risk analysis in a real-world environment
Source: PLOS Digit Health. 2025 Nov 13;4(11):e0001053. doi: 10.1371/journal.pdig.0001053 (PMC12614517; doi:10.1371/journal.pdig.0001053)

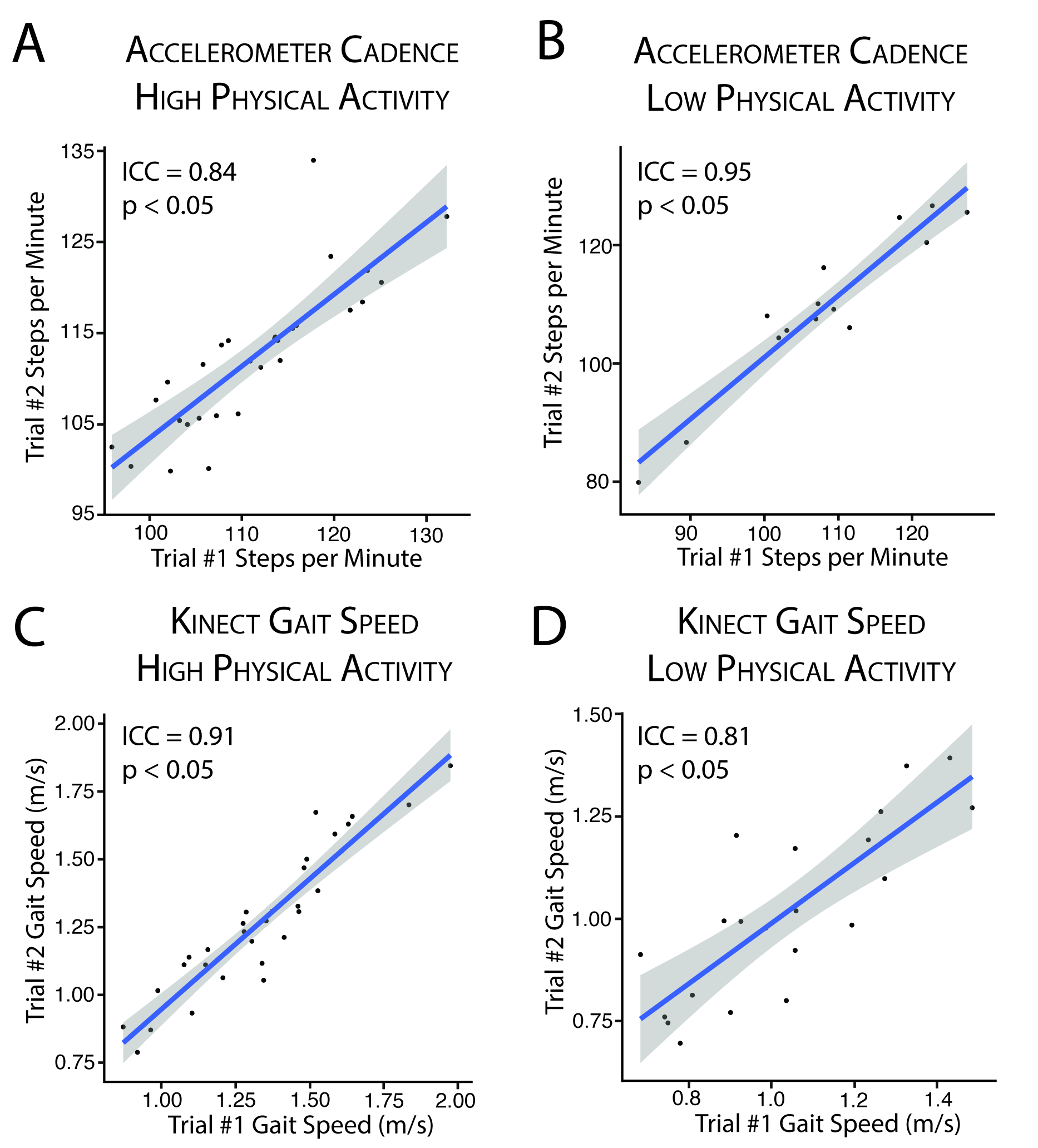

Supplement: S1 Fig — Trial #1 and Trial #2 at regular walking pace showed high intra-class correlation coefficients for cadence and gait for patients with high physical activity (Panels A and C, self-report frequent energetic physical activity >3 times per week) as well as patients who have low physical activity (Panels B and D, self-report frequent energetic physical activity hardly ever/never). All intra-class correlation coefficient p values were < 0.05. (TIF) [file pdig.0001053.s001.tif]

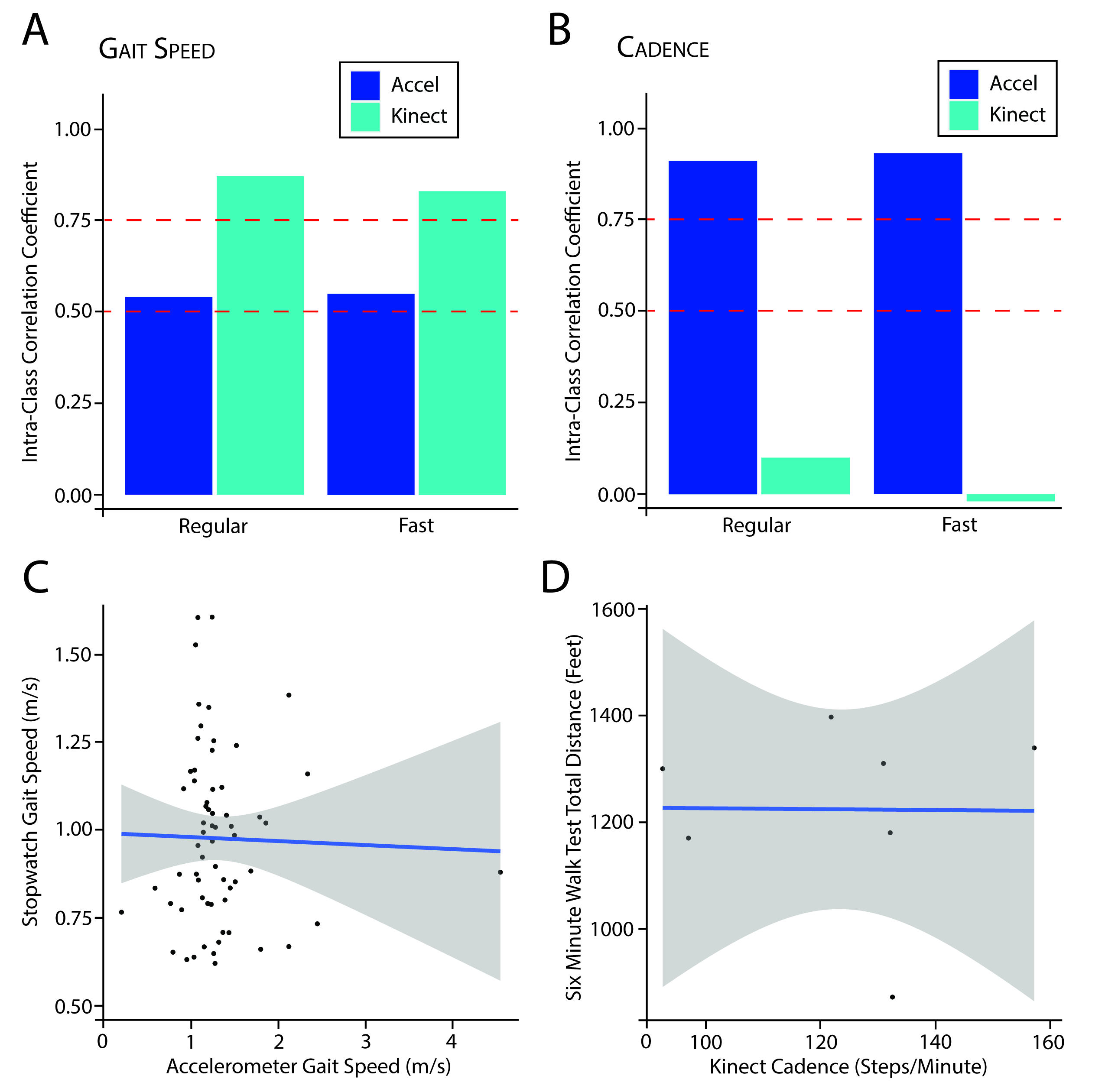

Supplement: S2 Fig — (A) Kinect-derived gait speed has higher intra-class correlation coefficients at both regular and fast pace than accelerometer-derived gait speed. (B) Accelerometer-derived cadence has higher intra-class correlation coefficients at both regular and fast pace than Kinect-derived cadence. Red dotted lines mark the thresholds for poor (< 0.5), moderate (0.5-0.75), and high (>0.75) intra-class correlation. (C) Accelerometer-derived gait speed is not associated with stopwatch-derived gait speed (p > 0.05). (D) Kinect-derived cadence is not associated with six minute walk test distance (p > 0.05). (TIF) [file pdig.0001053.s002.tif]
